# Evaluating compliance to a low glycaemic index (GI) diet in women with polycystic ovary syndrome (PCOS)

**DOI:** 10.1186/1756-0500-4-53

**Published:** 2011-03-08

**Authors:** Nicola Egan, Anna Read, Paddy Riley, William Atiomo

**Affiliations:** 1 Nottingham Medical school, University of Nottingham D Floor, East Block, Queens Medical Centre Campus Nottingham University Hospitals, Nottingham NG7 2UH, UK; 2 Department of Dietetics and Nutrition, Queens Medical Centre Campus, Nottingham University Hospitals, Nottingham NG7 2UH, UK; 3 School of Clinical Sciences, University of Nottingham D Floor, East Block, Queens Medical Centre Campus Nottingham University Hospitals, Nottingham NG7 2UH, UK; 4 Division of Human Development, School of Clinical Sciences, University of Nottingham D Floor, East Block, Queens Medical Centre Campus Nottingham University Hospitals, Nottingham NG7 2UH, UK

## Abstract

**Background:**

A low Glycaemic Index (GI) diet may decrease some long-term health risks in Polycystic Ovary Syndrome (PCOS) such as endometrial cancer. This study was performed to assess compliance to a low GI diet in women with PCOS. Food diaries prospectively collected over 6 months from women on a low GI diet or healthy eating diet were analysed retrospectively. The women were recruited for a pilot randomised control trial investigating whether a low GI diet decreased the risk of Endometrial Cancer. Nine women with PCOS completed 33 food diaries (17 from women on a low GI diet and 16 from women on a healthy eating diet) recording 3023 food items (low GI group:n = 1457; healthy eating group:n = 1566). Data was analysed using Foster-Powell international values inserted into an SPSS database as no scientifically valid established nutrition software was found. The main outcome measures were mean item GI and Glyacemic Load (GL), mean meal GL, percentage high GI foods and mean weight loss.

**Findings:**

Women allocated the low GI diet had a statistically significant lower GI of food items (33.67 vs 36.91, p < 0.05), lower percentage of high GI foods (4.3% vs 12.1%, p < 0.05) and lower GL of food items and meals.

**Conclusion:**

Women with PCOS on a low GI diet consumed food items with a significantly lower mean GI and GL compared to the healthy eating diet group. Longer term compliance needs evaluation in subsequent studies to ascertain that this translates to reduced long term health risks.

**Trial Registration:**

ISRCTN: ISRCTN86420258

## Background

Polycystic Ovary Syndrome (PCOS) is a complex heterogeneous condition affecting 5-10% of women of reproductive age in the UK [1-5]. The clinical problems include infertility, oligomenorrhoea, obesity and hirsutism and longer term health risks include diabetes, endometrial cancer, and increased cardiovascular morbidity [6-9]. It is thought that insulin resistance is central to the pathophysiology of PCOS [10-12], which underpins the rationale for measures that improve insulin resistance such as dietary modification, exercise and the use of Metformin in the treatment of PCOS and prevention of the long term health risks.

It has, been suggested that dietary modification using a low calorie low glycaemic index (GI) diet could specifically reduce some of the health risks associated with PCOS including endometrial cancer when compared to other diets [13-15]. A low GI diet contains carbohydrates that minimise changes in post prandial glucose levels and leads to a sustained reduction in hyperinsulinaemia [16]. However the realisation of any long term benefits requires compliance to the low GI diet. This study assessed compliance to a low GI diet in women with PCOS using food diaries collected prospectively over six months as part of a pilot randomised control trial at Nottingham University Hospital investigating whether a low GI diet decreased the risk of endometrial cancer [17]. Women had been asked to complete weekly food diaries on week one and months one, three & six. The objectives of the compliance sub analysis were to:

1. Assess the current methods of measuring compliance to a low GI diet and determine the most effective way for use with food diaries.

2. Measure compliance to a low GI diet for women on a low GI 600 kcal deficit diet comparing the proportion of GI foods in the diet of this group to foods eaten by women on a healthy eating 600 kcal deficit diet.

3. Determine whether there was any decrease in compliance over the course of the six month study.

## Methods

This study was conducted according to the guidelines laid down in the Declaration of Helsinki and all procedures involving human subjects/patients were approved by the Derbyshire Research Ethics Committee. REC reference number: 06/Q2401/76. Written informed consent was obtained from all subjects.

Details of the methods used in the randomised controlled trial have been previously published [17] but briefly; women had been recruited from gynaecological clinics at the Queens Medical Centre in Nottingham, and also a dietician running a regular PCOS weight management clinic. Volunteers were also requested from a PCOS website (http://www.pcos.i8.com) and posters displayed in the Queens Medical Centre. The trial entry criteria were: an objective diagnosis of Polycystic Ovary Syndrome using the Rotterdam criteria, oligo or amenorrhoea, age above 35, and a body mass index (BMI) calculated as weight in kilograms/height in metres squared, above 30. Exclusion criteria included previous or current history of any cancer, use of the combined pill, progesterones or clomiphene, women about to undergo intrauterine insemination and in-vitro fertilisation. Women from the clinics and volunteers from other sources were invited to make contact to arrange further assessment. One thousand four hundred and thirty three new and 2598 follow up patients were seen in 153 gynaecology clinics over 12 months. Of these, 441 (11%) potentially eligible women were identified. Nineteen patients were identified who met the trial criteria of which 11 were recruited to the trial and were randomised by a web based programme; six to a 600 kcal deficit low GI diet and five to 600 kcal deficit hypocaloric healthy eating approach. The numbers allocated to each arm and randomisation groups were unknown to the food diary analyst until data had been entered therefore single blinding the study.

The women completed food diaries at the start of the study and then at one, three and six month stages. Baseline dietetic advice and information was provided and ongoing support offered before the completion of these diaries. The senior dietician involved in the study explained the protocol and distributed personal record booklets providing information for patients depending on the diet plan to which they were randomised. The booklets contained information on the particular diet, together with an appointment progress record and test results. Patients were told to follow the diet as closely as possible every day for six months and keep the food diaries with as much detail as possible. The diaries emphasised the importance of portion sizes and eating breakfast, lunch and an evening meal every day. Snacking was allowed within the diet from a daily allowance totalling 250 kcal from a selection provided. These additional calories were included in the diet regimen. At least three and a half hours of exercise a week was recommended, but no upper limit was set.

The primary outcome measure was the mean GI of food items consumed as recorded in the food diaries. Secondary outcome measures included the proportion of foods consumed with a low GI, the mean Glycaemic Loads (GL) of food items and meals consumed, and changes in weight, and BMI.

A valid method of assessing compliance to a low GI diet though food diaries was sought using an internet search engine (Google), a literature search (Medline 1996 -present, Embase, CINAHL, BNI, Athens and Cochrane) and personal enquires from international and local dietetic experts (Personal communication; discussions took place with: Nottingham university (Moira Taylor, Kirsten Whitehead, Arlene Barton); University of Glasgow (Nigel Denby); University of Surrey (Gary Frost), Diabetes consultant; Clorado (David Mendosa); University of Sidney (Jennie Brand Miller, Alan Barclay) and University of Toronto (Thomas Wolever)). Enquiries suggested that there was no scientifically valid established nutrition software available to accurately analyse the GI and GL of dietary intake. Therefore an SPSS database was created populated by internationally accepted GI values from the Foster-Powell [16] international table of GI and GL values which combined all relevant data published between 1981 and 2001 and included GI values for over 750 types of foods, with nearly 1300 separate entries. For the various foods consumed in the diaries, the best matched GI value was assigned by manually reviewing the table which has been used in many published studies [13,15,18-21], and was recommended by the experts contacted. If there were GI values for multiple brands of the same food, the average value was taken. In cases where foods did not correspond to food types in published values, the GI was left absent. However foods containing little or no carbohydrate (such as meat, poultry, fish, salad vegetables or eggs) were assumed to be zero.

The mean and median GI of food items, proportion of high GI foods used and GL of food items and meals were compared between women randomised to a low GI diet compared to the healthy eating diet. Means were compared using the independent Students t-test, medians using the Mann-Whitney test and proportions using Chi squared. The difference in proportion of low medium and high GI foods between the two groups was assessed by Chi squared and the One way Analysis of Variance (ANOVA) test was used to discover whether there was a significant trend of mean GI and GL over the six month period, supplemented by a multiple range test (if the ANOVA was significant) to identify particular differences. Finally the accuracy of a commercially available software package (NutriGenie [22]) was analysed by comparing results generated using it with results generated using the SPSS package and measuring agreement using the Kappa test. A p value of <0.05 was considered statistically significant.

## Results

There was no significant difference in the clinical or biochemical the features in the women with PCOS entered into the either arm of the trial [17]. Overall, 33 of a possible 44 food diaries (75%) were completed by nine of the 11 women originally randomised. 17 diaries were from women randomised to a low GI diet and 16 diaries from women on a healthy eating diet. Three thousand and twenty three food items were recorded in these diaries (n = 1457; low GI group and n = 1566 healthy eating group). Table 1 shows a summary of the completeness of data collection. There was no significant difference between the two arms in the number of diaries completed or clinic attendance as shown in table 2.

**Table 1:** Summary from all subjects of the results of the completeness of data collection for dietetic intervention

Participant number	Number of monthly diet clinics attended	Number of food diaries completed	Was the participant considered to complete the study?
**1**	5	4	Yes

**2**	4	4	Yes

**3**	6	4	Yes

**4**	5	3	Yes

**5**	6	4	Yes

**6**	6	4	Yes

**7**	**3**	**2**	**Dropped out**

**8**	5	4	Yes

**9**	**0**	**0**	**Dropped out**

**10**	6	4	Yes

**11**	**0**	**0**	**Dropped out**

**Table 2:** A comparison of completed food diaries and clinic attendance from all subjects for each study arm

	Women randomised to 600 kcal deficit Low GI diet (n = 6)	Women randomised to 600 kcal deficit Healthy Eating diet (n = 5)
Number of diet clinics attended (% of total possible attendances)*	24 (66.7%)	22 (73.3%)

Number of food diaries completed (% of total possible diaries)*	17 (70.8%)	16 (80.0%)

**Number of food items recorded in the diaries Week 1**	499	371

**Number of food items recorded in the diaries Month 1**	475	412

**Number of food items recorded in the diaries Month 3**	275	387

**Number of food items recorded in the diaries Month 6**	208	396

*Includes all people recruited to trial

The mean GI value in the group allocated a low GI diet was significantly lower than the healthy eating group (33.67 vs 36.91, p < 0.05), and the low GI group also had a significantly different distribution of GI foods in their diet with a higher percentage of low GI and medium GI foods (81.8% vs 74.6%) and a lower percentage of high GI foods (4.3% vs 12.1%, p < 0.05) (Table 3).

**Table 3:** Glycaemic Index of food recorded in food diaries from all subjects throughout the 6 month study

	Women Randomised to 600 kcal deficit low GI diet No. (%)	Women Randomised to 600 kcal deficit healthy eating diet No. (%)
**Total No. Items**	1457	1566

**Items with GI value present (from international database/assigned zero)**	1251 (85.9%)	1358 (86.7%)

Classification of foods*		

Low GI (55 or less)	889 (61.0%)	919 (58.7%)

Medium GI (56-69)	299 (20.5%)	249 (15.9%)

High GI (70+)	63 (4.3%)	190 (12.1%)

No value	206 (14.1%)	208 (13.3%)

Mean GI**	33.67	36.91

**Confidence Interval**	32.21-35.14	35.42-38.41

**Standard deviation of mean**	26.48	28.17

**Median**	38	46

*Significant difference using Chi squared (p < 0.0005)

**Significant difference using independent t-test (p = 0.003)

Table 4 shows the data across the six month trial period. The Mean GIs of the diets had been calculated at several time points after the diaries had been completed for a week following the baseline advice given by the dietician. It can be seen from table 4 that the mean GI calculated from the food diaries of women randomised to the low GI group was lower at all time points evaluated which was consistent with the fact that women randomised to a low GI diet consumed less GI food, thus complying with the original advice provided with the dietician provided at the start of the study. It however suggests that as time progressed, adherence to the low GI diet decreased. However, the significant difference was between 0 and three months only (ANOVA p = 0.02, 0-3 months p = 0.044) and there was no evidence of an overarching consistent trend with time. Table 5 shows the increasing GL of foods and meals consumed over the first three months of the trial although this did not continue into month six. Again there was a significant difference between 0 and three months only (ANOVA p = 0.019, 0-3 months p = 0.027) and there was no evidence of an overarching consistent trend with time.

**Table 4:** Summary of mean and median Glycaemic Index value of items and % high GI for the 6 month trial period from all subjects

	1 week	1 month	3 months	6 months	Total
**Mean GI low GI diet**	30.71	34.00	36.45	36.49	33.67

**Mean GI healthy eating diet**	33.75	37.08	37.30	39.53	36.91

**Median GI low GI diet**	37	38	44	48	38

**Median GI healthy eating diet**	38	46	44	48	46

**% High GI on low GI diet**	1.8%	5.1%	6.9%	5.3%	4.3%

**% High GI on healthy eating diet**	12.1%	10.4%	13.4%	12.6%	13.4%

**Table 5:** Summary of mean and median Glycaemic Load of food items and meals for the 6 month trial period from all subjects

	1 week	1 month	3 months	6 months	Total
**Mean item GL low GI diet**	6.64	8.58	9.65	8.89	8.15

**Mean item GL healthy eating diet**	8.60	9.15	10.18	10.94	9.81

**Median item GL low GI diet**	1.86	4.38	4.48	4.45	3.54

**Median item GL healthy eating diet**	2.06	4.48	4.53	6.86	4.48

**Mean meal GL low GI diet**	21.34	27.43	24.72	22.56	24.16

**Mean meal GL healthy eating diet**	26.86	29.90	31.00	33.73	30.40

**Median meal GL low GI diet**	18.55	22.55	23.45	18.83	20.28

**Median meal GL healthy eating diet**	24.40	25.36	28.53	25.34	26.79

A post hoc sample size calculation showed that for the difference in meal GI of food items in both groups found in our study (3.24), with a mean standard deviation of 27.32, the sample size required for an Alpha of 0.05 and a power of 80% was 1118 food items per group. In our study, 1457 food items were evaluated in the low GI group and 1566 in the healthy eating group.

Women in both groups lost weight and reduced their waist and hip circumference and BMI with a 5.37% reduction in mean weight over six months [17] and there was no significant difference between groups.

The accuracy of the NutriGenie software was analysed by comparing results generated with the results from analysis using the SPSS database. There were 293 food items in the diaries, 190 (64.8%) of which had an allocated GI value. These were categorised into low medium and high GI, using the internationally accepted criteria, to allow comparison with the NutriGenie database which had originally been considered to analyse the data. Only 107 items (36.7%) from the diaries were available on the NutriGenie database and 91 (31.1%) food items had both NutriGenie and Foster-Powell based SPSS database values. Table 6 compares the agreement between the NutriGenie and SPSS database where it can be seen that nine items classified as low in the SPSS database were classed as high in the NutriGenie. These and all other differences between the two databases were examined at source and the values on the SPSS database were checked back to the international database to ensure that there was no transcribing error. No transcribing errors were found although all items classified as low in the database had values bordering the medium classification. The poor agreement between methods was confirmed by a Kappa test value of 0.316 [23].

**Table 6:** Comparison of NutriGenie and SPSS database for 91 foods

		NutriGenie			Total
			
		Low	Medium	High	
**SPSS database**	**Low**	47	10	9	66
	
	**Medium**	3	0	12	15
	
	**High**	0	0	10	10

**Total**		50	10	31	91

## Discussion

This study showed that women with PCOS complied with a low GI diet. There was a significantly lower mean GI of food items and GL of food items and meals in women randomised to the low GI arms of the trial compared to the healthy eating arm. The results suggested that compliance decreased as the study progressed although the mean GI and GL of food items and GL of meals were lower at all stages in the low GI group compared to the healthy eating group. The average GI of food items was 8.8% lower in the low GI group. The proportion of low, medium and high GI foods also differed significantly between the two arms, and the intake of high GI foods was lower in the low GI group. As far as we know, this was the first study to have assessed compliance to a low GI diet by analysing the GI value of data prospectively collected in food diaries and there were no published studies to compare the findings with.

The study was limited by various factors. A key limitation was the small sample size but although the study had a small number of women entered, the majority of diaries were well completed and the data available were large, so overall statistically significant differences were observable. However trends and sub group analysis were not statistically significant due to the small sample size. Although selection bias was limited due to randomisation, the small sample size may have increased the potential effect from volunteer bias and non-participation bias. Of those volunteering or referred to the trial only 19 met all eligibility criteria, 11 entered the trial and nine completed the trial. It is more likely that women who dropped out of the trial would not have complied with the dietary intervention, increasing the chance of the results showing compliance. However, a strength of the study was that it was linked to a rigorously conducted CRUK pilot which had consistent entry criteria, thorough randomisation, and good dietetic support for participants. The diaries were set out in a way encouraging a high level of detail, potentially allowing all food and drink consumed each day with quantities to be recorded. Printed recording booklets for food intake prompted patients for the desired information and structured data in an organised way facilitating data analysis. This assessment method, when completed properly, was a robust way of gathering data and has been shown to have a beneficial reactivity effect [24] increasing compliance.

Another limitation was the lack of universal agreement on the GI values of foods, whether drinks such as tea and coffee should be included and the complexities around how to account for issues such as ripeness of fruit and specific combinations of foods which potentially affect each other. No account of food interactions was included in the analysis. The inclusion of standard portion sizes could have introduced inaccuracy but this will not have affected the results related to GI of food items recorded.

Although this study was single blinded, information bias could have occurred. The dietician knew which study arm patients were allocated to and more importantly the patients knew what intervention they were having in terms of low GI or healthy eating diet. In addition, the self monitoring by patients meant that control of data collection was the patient's full responsibility so the accuracy of the data relied on the patient's compliance to keeping the diary. The potential for bias in self completed diaries where the individuals knew what intervention they should be following was high. Less desirable eating episodes may have been excluded from the diaries, biasing the monitored behaviour in the desired direction. There was also the possibility of recall bias where information may have been entered retrospectively from memory leading to inaccurate recordings. The Hawthorne effect could have introduced bias during the whole study but particularly during the four weeks out of the six month trial that patients were required to fill in a food diary and the diaries may not have been representative of the other 20 weeks the patients were expected to comply with the dietetic advice. The direction of these biases would be to increase the likelihood of finding compliance to a low GI diet but the five percent weight loss in both arms of the trial suggests that the calorie deficit and/or exercise component was complied with. However it is not possible to totally rule out the Hawthorne effect in behaviourally based studies or to truly blind the participants to dietetic interventions.

The internationally accepted range for low GI intake is 0-55 and both groups in the study had an average GI of food items and GL of foods that was low. The average GI for items was 33.67 for the low GI arm and 36.91 for the healthy eating group. The average GL of items for the low GI diet was 8.15 and 9.81 for those on the healthy eating diet and it is suggested that the GL of items is low when under or equal to the value of 10 [25]. These effects may be the result of the general advice and information given by the dieticians as many of the healthy eating diet foods suggested, such as salads, fruit and vegetables were similar to those suggested for the low GI diet and usually have a low GI. The main high GI foods within the diaries were potato and certain breads and breakfast cereals of which the participants of the low GI diet were advised to avoid in the personal record booklet suggesting all participants followed dietetic advice.

After enquires to find an appropriate programme to assess the food diaries it became apparent that an affordable commercial database was not available. The NutriGenie software initially looked a possible solution for qualitative analysis of whether the diet was predominantly low GI. However there was poor agreement of food classification when comparing NutriGenie and the SPSS database which used nationally published and accepted values (Kappa = 0.316). NutriGenie, despite claiming thousands of entries, contained significantly fewer foods from the diaries. It is not surprising that the commercial programme contained less relevant foods than the SPSS database as the latter was purpose made, but the difference in allocation of a food group to low, medium and high was surprising. The company marketing the NutriGenie software was reticent in giving information about sources of information for their database, but this is an illustration of the complexity of analysing the GI of diets accurately and also gives an understanding of the view of some health professionals that it is too complex to be a basis for dietary intervention.

This study, although small, is of interest due to the potential benefits of a low GI diet for the treatment of PCOS associated diabetes and obesity and the increasingly strong suggestion that the endocrine and metabolic abnormalities present in PCOS produce an association with endometrial cancer [1,2,5,9].

A low GI diet where reduction of insulin levels lowers testosterone levels, improves hirsutism and acne, improves menstrual function, dislipidaemia and potentially decreases the risk of endometrial cancer [1,11,13-15] has led to support for its use in both obese and lean patients with PCOS [26]. Realisation of any long term benefits such as cancer prevention would require compliance to the low GI diet. This study suggests that compliance is possible over a six month period, although longer term compliance would still need to be assessed. The benefits of lifestyle intervention in diabetics [27] show that a slightly restricted but healthy lifestyle can reduce long term health problems linked with insulin resistance and this study suggests that it might be possible for women with PCOS.

## Conclusions

Women with PCOS allocated to a low GI diet consumed food items with a significantly lower mean GI and GL compared to the healthy eating diet group. However, challenges in accurately assessing compliance to a GI diet were identified, particularly the impracticality of the dietician and participants being blind to the arm of the trial. This and various other confounding variables were likely to have worked in the direction of increasing the likelihood of finding compliance to the dietetic advice although unlikely to remove the differences found between the two groups. This study gives a degree of confidence, but no absolute confidence, that if a full study to determine the effects of low GI diet on women with PCOS takes place it is likely the intervention will be complied with. Such a trial will give the opportunity for longer term compliance to be assessed. The benefits of lifestyle intervention in people with diabetes show that it is possible for dietary modification and exercise aimed at achieving a weight loss of 5-10% to reduce the risk of long term health problems linked with insulin resistance and it is important to confirm this finding in women with PCOS.

## Competing interests

The authors declare that they have no competing interests.

## Authors' contributions

NE summarised and analysed the data in the food diaries, wrote up and edited the drafts of the paper for publication. AR provided the dietetic advice to the participants, supervised the collection of data contained in the food diaries and edited the drafts of the paper for publication. PR provided statistical support and edited the drafts of the paper for publication. WA conceived of the idea, organised and ran the primary randomised controlled trial, supervised the collection of data, data analysis and edited the drafts of the paper for publication. All authors read and approved the final manuscript.
